# Tantalum Oxynitride Thin Films: Assessment of the Photocatalytic Efficiency and Antimicrobial Capacity

**DOI:** 10.3390/nano9030476

**Published:** 2019-03-23

**Authors:** Daniel Cristea, Luis Cunha, Camelia Gabor, Ioana Ghiuta, Catalin Croitoru, Alexandru Marin, Laura Velicu, Alexandra Besleaga, Bogdan Vasile

**Affiliations:** 1 Materials Science and Engineering Faculty, Transilvania University, Eroilor 29, 500036 Brașov, Romania; daniel.cristea@unitbv.ro (D.C.); camelia.gabor@unitbv.ro (C.G.); ioana.ghiuta@unitbv.ro (I.G.); 2 Physics Center, Minho University, Gualtar Campus, 4710-057 Braga, Portugal; lcunha@fisica.uminho.pt; 3 Institute for Nuclear Research Pitesti, Str. Campului Nr. 1, POB 78, 115400 Mioveni, Arges, Romania; marin.alexandru.horia@gmail.com; 4 Faculty of Physics, Alexandru Ioan Cuza University, 11 Carol I Blvd, 700506 Iasi, Romania; velicu.laura@yahoo.com (L.V.); alexandra_besleaga@yahoo.com (A.B.); 5 University Politehnica of Bucharest, National Research Center for Micro and Nanomaterials, Gh. Polizu Street No.1-7, 011061 Bucharest, Romania; vasile_bogdan_stefan@yahoo.com

**Keywords:** tantalum oxynitride, magnetron sputtering, surface roughness, photocatalytic activity, antibiofilm capacity

## Abstract

Tantalum oxynitride thin films have been deposited by reactive magnetron sputtering, using a fixed proportion reactive gas mixture (85% N_2_ + 15% O_2_). To produce the films, the partial pressure of the mixture in the working atmosphere was varied. The characteristics of the produced films were analyzed from three main perspectives and correspondent correlations: the study of the bonding states in the films, the efficiency of photo-degradation, and the antibacterial/antibiofilm capacity of the coatings against *Salmonella*. X-ray Photoelectron Spectroscopy results suggest that nitride and oxynitride features agree with a constant behavior relative to the tantalum chemistry. The coatings deposited with a higher reactive gas mixture partial pressure exhibit a significantly better antibiofilm capacity. Favorable antibacterial resistance was correlated with the presence of dominant oxynitride contributions. The photocatalytic ability of the deposited films was assessed by measuring the level of degradation of an aqueous solution containing methyl orange, with or without the addition of H_2_O_2_, under UV or VIS irradiation. Degradation efficiencies as high as 82% have been obtained, suggesting that tantalum oxynitride films, obtained in certain configurations, are promising materials for the photodegradation of organic pollutants (dyes).

## 1. Introduction

Transitional metal oxynitride thin solid films have found a wide application in industrial environments, due to interesting electrical, optical, mechanical, and other properties in association with a relatively low cost and ease of manufacturing. Films from this class of compounds (oxynitrides) are promising candidates to be implemented in practical applications, due to their main feature, which is the possibility to tune firstly, the metallic/non-metallic ratio, and secondly, the non-metallic content ratio (O/N), by closely controlling the deposition parameters. Tantalum oxynitride thin films have already been studied for their potential in tribological and/or mechanical applications [1,2], electrical applications [3,4,5,6,7,8], and optical applications [9,10]. 

One promising direction for the tantalum oxynitride film system is that of materials with photocatalytic properties. Several reports can be found in the literature, certifying their potential for this type of application, under ultraviolet radiation, but more importantly, under visible radiation. However, to the best of our knowledge, only a limited number of reports are related to tantalum oxynitrides under thin film form. As reported by Hsieh et al. [11,12], a nitridation and oxidation process, implemented on the pure tantalum film, prepared by DC magnetron sputtering, was used to create tantalum oxynitride films at elevated temperatures and different oxygen flow rates. The photocatalytic activity of the obtained tantalum oxynitride films was investigated by the photodegradation test under visible light. In this case, the decomposition of methylene blue on tantalum oxynitrides under visible light (λ = 610 nm) irradiation, leading to the reduction of optical absorbance, was observed. The photodegradation of methylene blue was presented in [13], and it was observed that the co-deposition of Ag in TaO_x_N_y_ films can enhance the photocatalytic behavior of the composite films. The reason for this increased photodegradation capacity is thought to be due to the increased number of electron–hole pairs [14]. Another report [15] mentions the deposition of TaO_x_N_y_ photo-electrodes by DC reactive sputtering at room temperature. The authors motivate the nitrogen or oxygen incorporation within oxides or nitrides in order to modify the band gap of the starting material, so that the resulting band gap will exhibit optimal absorption properties, in relation to the requirements of the application. In the same report, it is mentioned that an increase in the oxygen partial pressure affects the maximum absorption domain, which is shifted to lower wavelengths for higher oxygen partial pressures. Dabirian et al. [16] report the manufacture of stable TaO_x_N_y_ films, obtained in several stages: first, the deposition of a tantalum layer on top of a titanium layer (its role: adhesion layer), followed by an oxidation period of 5 h at 650 °C in air and a subsequent nitridation stage. The photocatalytic capacity was assessed by photoelectrochemical (PEC) experiments, performed under AM 1.5 illumination. The authors suggest that higher nitridation temperatures can be expected to give lower defect concentrations and higher photocurrents during PEC experiments. A comparison of TaO_x_N_y_ and Ta_3_N_5_ [17], regarding their capacity to degrade atrazine, revealed that TaO_x_N_y_ samples were more efficient than Ta_3_N_5_ samples. This higher photocatalytic activity of TaO_x_N_y_ was explained by a higher separation efficiency of electrons and holes of TaO_x_N_y_, which could be related to the larger specific surface area. Furthermore, it was reported [18] that under visible light irradiation (420 nm ≤ λ ≤ 700 nm), tantalum (oxy)nitrides can oxidize water to O_2_ and reduce H^+^ to H_2_ in the presence of sacrificial reagents (Ag^+^ and methanol).

The excellent corrosion resistance, fracture toughness, and biocompatibility of tantalum-based materials recommend their use as biomaterials in several applications: vascular clips, flexible vascular stents, and dental implants, as well as orthopedic components, as acetabular cups, or other trabecular metal components [19,20].

Bulk Ta is highly unreactive and biocompatible in the body. Ta does not exhibit toxicity to surrounding cells or inhibit local cell growth of surrounding bone. Moreover, it was found that pure tantalum exhibits a lower or similar *S. aureus* and *S. epidermidis* adhesion when compared with commonly used materials in orthopedic implants [21].

The biocompatibility, antibacterial properties, and cytocompatibility characteristics of tantalum-based coatings (mostly oxides) are presented, to date, in a limited number of references. Chang et al. [22] report the deposition of Ta and amorphous tantalum oxide coatings by magnetron sputtering. A hydrophilic crystalline β-Ta_2_O_5_ coating was obtained by rapid thermal annealing of the deposited tantalum oxide coating at 700 °C. It was observed that the antibacterial effect is more obvious for the deposited amorphous Ta_2_O_5_ coating than for the uncoated and other coated specimens. Moreover, Huang et al. [23] reported that tantalum nitride (TaN) coatings with a hydrophobic surface (contact angle = 92.0 ± 1.2°, mean ± SD) exert antibacterial effects against *Staphylococcus aureus*. 

To the best of our knowledge, to date, there are no reports presenting results concerning the antibacterial/antibiofilm capacity of tantalum oxynitride materials, either in bulk, powder, or coating form. This work presents our results concerning the dye photodegradation and antibiofilm capacity of magnetron sputtered tantalum oxynitride thin films, corelated to the surface morphology, bonding states, and structural features.

## 2. Materials and Methods 

### 2.1. Sample Preparation

TaO_x_N_y_ thin films were deposited on silicon (100) wafers, stainless steel (AISI 316L), and glass substrates by DC reactive magnetron sputtering, using a laboratory-size deposition chamber, containing a tantalum target (99.6% purity), with the dimensions 200 × 100 × 6 mm, positioned at 70 mm relative to the substrate holder. The gas atmosphere during deposition was composed of argon as the working gas and a mixture of nitrogen and oxygen (fixed proportion of 15% O_2_ + 85% N_2_) as reactive gases. The argon flow during deposition (70 sccm) was kept constant during all depositions, while the overall N_2_ + O_2_ gas mixture flow was varied between 2.5 and 30 sccm, corresponding to partial pressures ranging from 0.02 Pa to 0.24 Pa (registered before the plasma ignition), according to Table 1. 

The substrate holder was electrically grounded (GND), and the temperature was kept at 100 °C for all depositions. A more detailed presentation for the deposition protocol for the samples included in this work is presented elsewhere [24]. 

### 2.2. Structural and Morphological Characterization

X-ray Photoelectron Spectroscopy (XPS) measurements were performed on silicon substrate samples, with an Escalab 250 system (Thermo Scientific, East Grinstead, United Kingdom) equipped with a monochromated Al K_α_ (1486.6 eV) X-ray source and a 10^−8^ Pa base pressure in the analysis chamber. The acquired spectra were calibrated with respect to the C1s line of surface adventitious carbon at 284.8 eV. An electron flood gun has been used to compensate for the charging effect in insulating samples.

A gentle removal of the contaminants confined on the outermost surface layers of the thin films was performed prior to the XPS surface chemical analysis. An Ar^+^ ion gun operated with a low energy ion beam of 1.0 keV was rastered over a (3 × 3) mm^2^ area and a 0.5 min sputter time was used for surface etching.

The reflectance of the coatings deposited on silicon was measured on a UV-Vis-NIR spectrophotometer (Shimadzu UV-Vis-NIR 2505) in the spectral range of 250-800 nm. These measurements were used to find the type of energy transition and the optical band gap of the Ta-based phases (Tauc plots).

Transmission electron microscopy (TEM) analyses were performed on selected samples (coatings deposited on silicon wafers, prepared by erosion of the substrate) using a Tecnai G2 F30 STWIN electron microscope from Thermo Fisher Scientific (Former FEI) operated at an acceleration voltage of 300 kV. The images were obtained in normal BFTEM (Bright Field Transmission Electron Microscopy) mode and HRTEM (High Resolution Transmission Electron Microscopy) mode.

Topological characteristics of both coated and uncoated glass samples were analyzed in terms of root mean square (RMS) surface roughness, by Atomic Force Microscopy (AFM), using an NT-MDT Solver Pro-M system. All the measurements were performed in non-contact mode, under ambient conditions, over multiple random (10 × 10) μm^2^ areas.

### 2.3. Photoactivity Testing

The photodegradation behavior of the TaO_x_N_y_ thin films was tested using two model azo-dyes, namely anionic methyl orange (C_14_H_14_N_3_SO_3_Na) (MO) and cationic methylene blue (C_16_H_18_N_3_SCl) (MB).

The reason for choosing methyl orange as the photodegradation medium is represented by its high stability to degradation in comparison with other model dyes, such as methylene blue, thus allowing the photoefficiency response of the thin films to persistent organic pollutants to be simulated. Methylene blue, on the other hand, is often used in the reference literature to assess the type of photodegradation kinetic for various photocatalysts, especially under visible light irradiation conditions [25]. The MO photodegradation ability of the eight different TaO_x_N_y_ thin films deposited on glass substrates (according to Table 1), presenting a photoactive area of 2.5 cm^2^, was assessed under ultraviolet (UV) and visible (VIS) irradiation conditions by using an open-air photodegradation reactor. The uncoated glass substrate (without any deposited film) has been used as the reference. 

For the UV irradiation experiments, three F18W/T8 black light tubes (Philips) (UVA light, typically 340–400 nm, with peak emission at λ_max_ = 365 nm), placed annularly in relation to the photoreactor stand, were used for photolysis. For the VIS irradiation experiments, three TLD Super 80 18 W/865 white light tubes (Philips, λ > 400 nm) were used, annular to the photoreactor stand. The thin film samples and glass substrate reference were submerged in 25 mL of dye solution (MO and MB, respectively), with an initial concentration of c_0_ = 0.0125 mM, ensuring a constant irradiation power density of 60 mW/cm^2^.

Before starting the photocatalysis experiments, the films were firstly equilibrated in the dye solution in the dark for 5 min, to ensure an adsorption-desorption equilibrium of MO or MB on the surface of the samples. 

The photo-response of the sputtered samples using MO as the model dye was evaluated under four different conditions: (i) ultraviolet (UV) irradiation; (ii) UV irradiation with 100 μL of 30% wt. H_2_O_2_ added to the volume of MO solution in each flask; (iii) visible (VIS) irradiation; and (iv) VIS irradiation with 100 μL of 30% wt. H_2_O_2_ added to the MO solution in each flask. The 30% wt. H_2_O_2_ solution was added as the oxidizing agent (electron acceptor) during the photocatalysis reactions. The effective H_2_O_2_ concentration in each flask, before photocatalysis, was 6.50 mg/L. This concentration was efficient in enhancing the MB removal rate, also providing an increased economic efficiency of the process (low amount of hydrogen peroxide), as found in other research [26,27].

The total duration of the photoirradiation experiments has been chosen at 6 h, considering that, at this period, photodegradation equilibrium occurs in the case of MO. The methyl orange removal efficiency (discoloration efficiency) *η* has been determined with the help of Equation (1), based on the absorption maxima for this dye (λ_max_ = 463 nm, determined with a Perkin Elmer Lambda 25 spectrophotometer):

(1) $$\eta =\frac{A_{0}-A}{A_{0}}\times 100$$

where *A*_0_ is the absorbance of the initial dye solutions in contact with the films, and *A* is the absorbance of dye solutions in contact with the TaO_x_N_y_ thin film samples, determined at λ_max_ = 463 nm, after the 6 h period of illumination. The same formula has been used to determine the removal efficiency for the reference samples.

Moreover, the photocatalytic activity of the TaO_x_N_y_ films deposited onto glass substrates in comparison with the reference (uncoated glass substrate) was evaluated by determining the removal efficiency and type of degradation kinetic for the MB dye under visible light irradiation conditions, without the addition of H_2_O_2_. The absorbances of the MB solutions at their absorption maxima (λ_max_ = 664 nm, measured with a Thermo Scientific Evolution 300 spectrophotometer) in contact with the samples were measured at determined time intervals until reaching equilibrium (total irradiation time of 2 h). The methylene blue removal efficiency at equilibrium was calculated with the help of Equation (1). 

The photoactive behavior of the TaO_x_N_y_ thin films and reference glass substrate was also evaluated by water contact angle measurements (*θ*), under UV irradiation. The measurements were conducted at room temperature, irradiating the samples by a UV lamp (Philips TUV T8) at a power density of 1.8 mW/cm^2^ and analyzing the height profiles of small sessile drops (1 µL) of deionized water, deposited on the sample surfaces, with the help of the ImageJ software. 

### 2.4. Antibiofilm Capacity Assay

The freeze-dried strain of *Salmonella* was recovered by culturing the bacteria in solid medium, containing 1 g/L yeast extract, 18 g/L agar-agar, 5 g/L sodium nitrate, and 0.2 g/L glucose, supplied by Scharlau Chemicals. The third generation of *Salmonella* strain was used for further studies. 

With a design to assay the antimicrobial/antibiofilm potential of the TaO_x_N_y_ coatings, 1 µL of *Salmonella* strain (1 × 10^−9^ CFU L^−1^) was inoculated into 50 mL liquid media, consisting of 0.6 g/L yeast extract, 1 g/L sodium nitrate, and 3 g/L glucose. The coated samples, deposited on stainless steel, and the control (uncoated stainless-steel disk, mirror polished), were placed in distinct Berzelius glasses. Thereafter, the samples were sterilized in an autoclave at 126 °C for 0.5 h to ensure their safe handling in antimicrobial testing.

A volume of 50 µL from the liquid suspension containing bacteria was placed on the surface of each sterile sample, followed by incubation at 33 °C for 48 h. After the incubation period, the samples were washed twice with PBS (phosphate-buffered saline), similarly with the method described by Liu et al. [28]. The specimens were subsequently submerged in PBS solution and then exposed to ultrasounds for 0.5 h, using an ultrasonic cleaner to detach the adherent bacteria from the surface of the samples. Simultaneously, Petri dishes containing solid media were prepared in order to inoculate the solution obtained through ultrasonication. The plates were incubated at 33 °C for 48 h, after which the inherent properties of tantalum in antibacterial/antibiofilm activity were evaluated by counting the bacterial colonies and assessing the area of dispersion [20,28]. The antimicrobial/antibiofilm assessment was carried out three times as a verification of the replicability of the experiment.

## 3. Results and Discussion

### 3.1. Spectral Characterization of the Thin Films

XPS was used to access the bonding state of atoms across the surface layers and, after quantitative analysis, to find the elements’ relative concentration and corresponding chemical states. 

**Figure 1:**
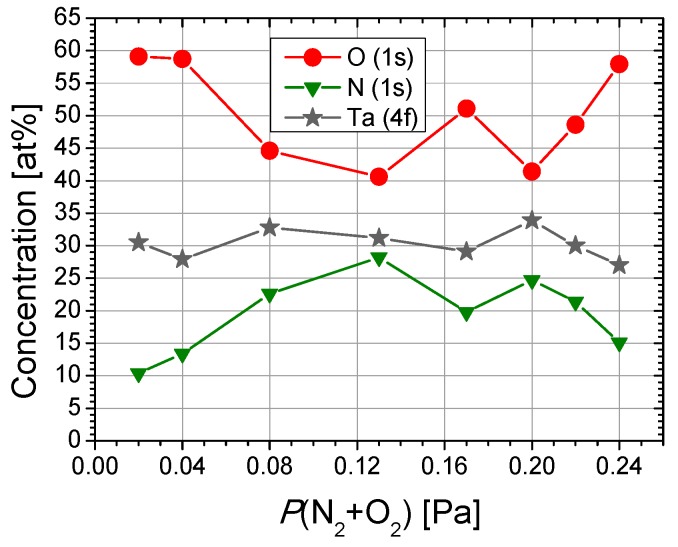
Atomic concentration of the films, as a function of the reactive mixture partial pressure, obtained from O (1s), N (1s), and Ta (4f) of XPS spectra.

XPS spectra revealed the characteristic XPS transitions of tantalum, oxygen, and nitrogen, proving the chemical interaction between metal atoms and the reactive gas mixture. No impurities or contaminants were detected in all samples analyzed in this study. The relative atomic concentration of the films, as a function of the partial pressure of the reactive gases (*P*(N_2_ + O_2_)), is depicted in Figure 1.

**Figure 2:**
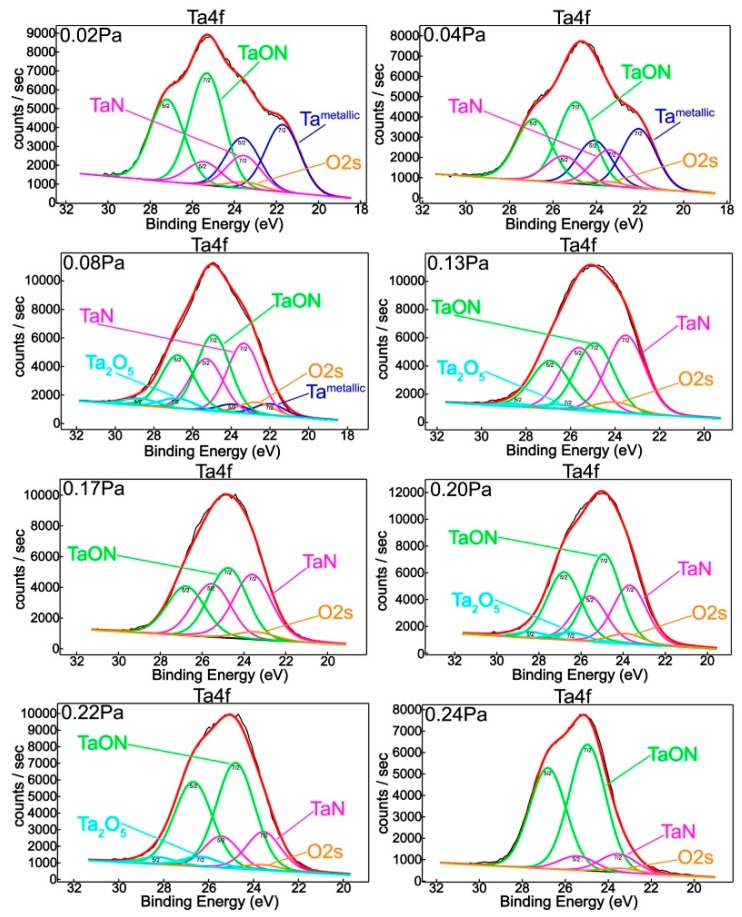
XPS deconvoluted Ta4f spectrum for samples obtained in different reactive gas mixture partial pressures.

To probe the surface chemistry of the films, O1s, N1s, and Ta4f XPS high resolution spectra were acquired. The surface chemical response of tantalum, as a function of the nitrogen/oxygen flow ratio, is portrayed in Figure 2. The tantalum chemical states were assessed after curve-fitting of the complex Ta4f envelops. Figure 1 summarizes the relative chemical concentrations. It is very important to mention that the data in the literature and databases are spread over a rather wide binding energy range.

The feature located at 22.0 eV can be ascribed to metallic Ta [7,29,30,31,32], while the peak at 23.5 eV corresponds to tantalum nitride [7,30,31,33,34,35]. The photoelectron binding energy peaks located at 25.0 eV and 26.5 eV can be attributed to tantalum oxynitride [35,36] and to the Ta^5+^ oxidation state corresponding to the tantalum pentoxide Ta_2_O_5_, respectively [29,30,32,33,37]. Chun and Ishihara reported 25.8 eV for the binding energy corresponding to tantalum oxynitride bonding, which is in close agreement with previous findings in the literature [7,32]. 

**Figure 3:**
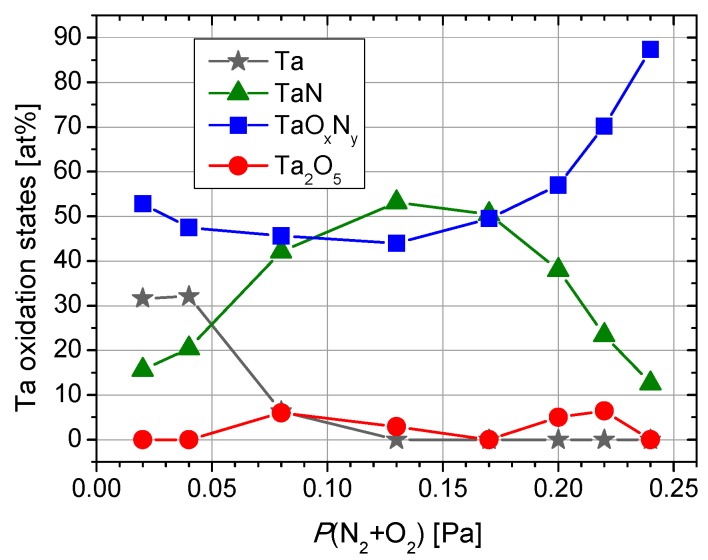
Tantalum chemical states relative concentrations based on the fitting process of Ta 4f XPS peak.

From Figure 3, one can notice a dramatic decrease of metallic Ta with the increase of the reactive gas mixture partial pressure, from ~32% to ~6% when *P*(N_2_ + O_2_) increases from 0.02 and 0.08 Pa, which completely vanishes for higher values of *P*(N_2_ + O_2_). This is a consequence of the increasing nitrogen relative concentration (Figure 1) in association with the corresponding increase of tantalum nitride content (Figure 2). Although the oxygen decreasing trend was accompanied by a decreasing oxynitride contribution, tantalum presents a fully oxidized state for 0.13 Pa and higher reactive gas partial pressures.

A completely opposite chemical behavior was observed in the case of films prepared with high partial pressure conditions. Thus, a decreasing tendency of nitride contribution followed by an increase of oxynitride feature was established by increasing *P*(N_2_ + O_2_) from 0.17 to 0.24 Pa, in good agreement with element relative concentrations (Figure 1). The peculiar chemical behavior of Ta_2_O_5_ can be explained by the occurrence of a threshold pressure value at 0.13 Pa in which diffusion/segregation processes take place that have an influence on the surface chemistry of deposited layers.

**Figure 4:**
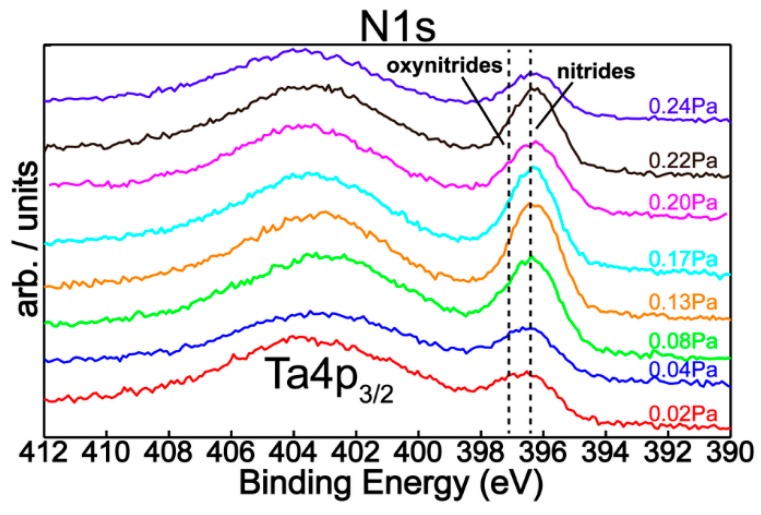
N1s XPS superimposed spectra for different reactive gas partial pressures.

**Figure 5:**
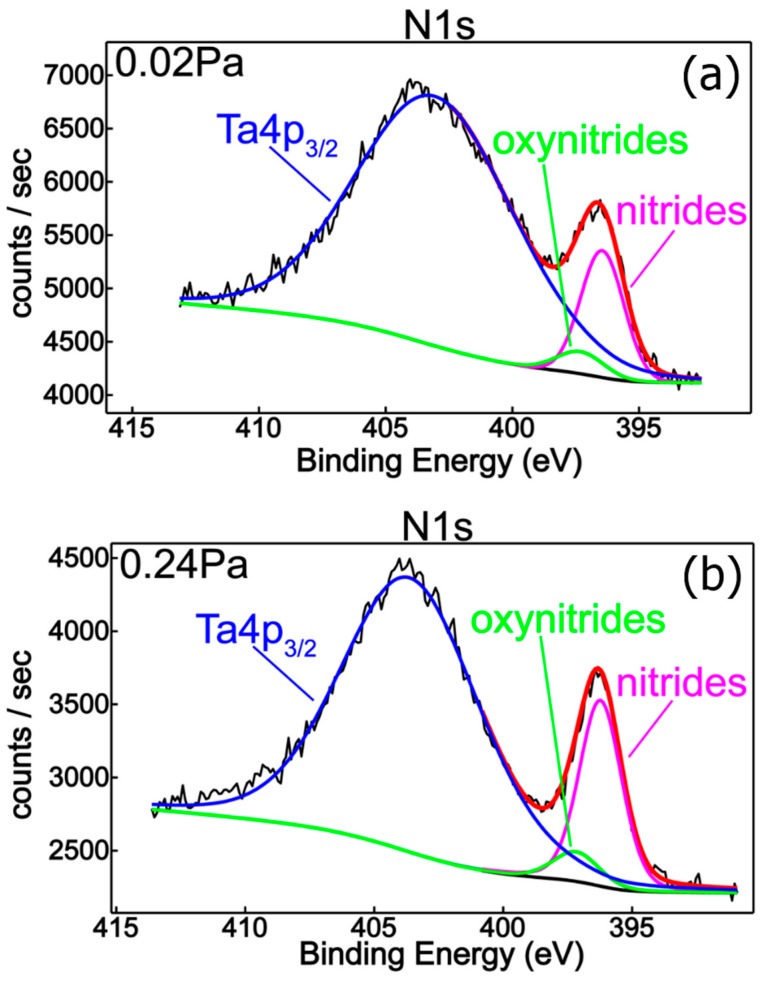
XPS deconvoluted N1s spectrum for samples obtained with different reactive gas mixture partial pressures: (**a**) 0.02 Pa; (**b**) 0.24 Pa.

**Figure 6:**
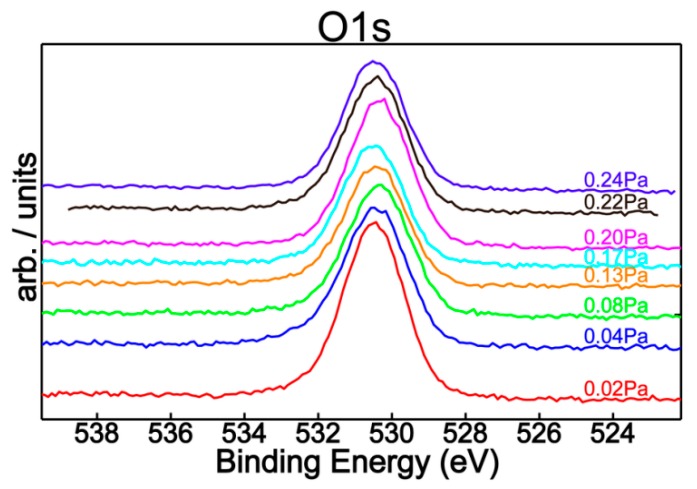
O1s XPS superimposed spectra for different reactive gas partial pressures.

Figure 4 shows the N1s superimposed core level spectra for all mixed reactive gas atmospheres, indicating a similar chemical behavior with small quantitative differences regarding the nitrogen content. In addition, spectral deconvolution of N1s (Figure 5) of the samples produced with *P*(N_2_ + O_2_) = 0.02 Pa and *P*(N_2_ + O_2_) = 0.24 Pa reinforce the assessment of metallic nitride and oxynitride films formation, in good agreement with tantalum chemistry (Figure 2). Therefore, the observed features in the nitrogen spectra are located at 396.5 eV and 397.2 eV, respectively, with the former being ascribed to metal nitrides [7,30,31,32,33] and the latter to metal oxynitrides [32]. Moreover, Figure 6 represents the O1s XPS superimposed spectra for different reactive gas partial pressures.

**Figure 7:**
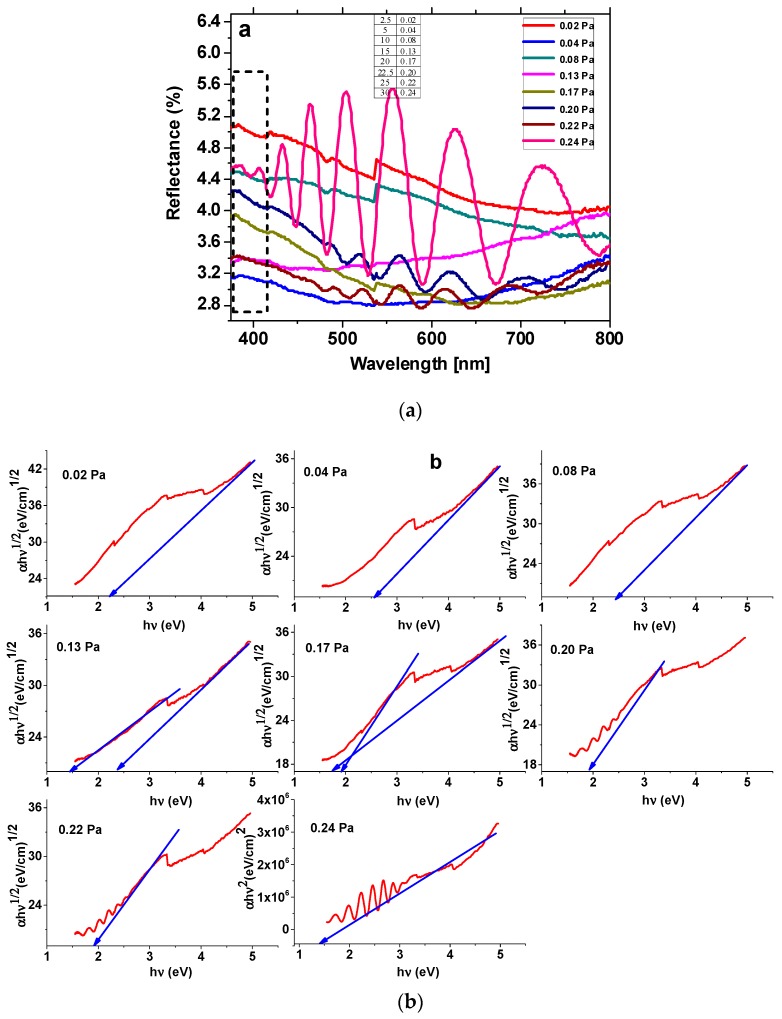
(**a**) UV-Vis spectra; (**b**) Tauc plots of the films.

The UV-VIS spectra of the films, shown in Figure 7, present a reflectance decrease (absorption) at ~408 nm, which could be attributed to a O^2−^ (2p) →Ta^5+^ (5d) charge transfer process [38]. These absorption maxima tend to shift to higher wavelengths as the partial pressure of reactive gases increases from *P*(N_2_ + O_2_) = 0.08 Pa, due to the presence of Ta_2_O_5_ on the surface of the samples. The samples containing the Ta (V) oxide, namely those obtained at partial pressures of 0.08, 0.13, 0.20, and 0.22 Pa, evidence higher reflectance values than the oxynitride-rich samples, as also determined in other research [12]. Another weak absorption mode can be evidenced at ~420 nm, which could be attributed to the N^V+^ (2p) → O^2−^(2p) orbital charge transfer process in TaON [39]. It is evident that this transition is prevalent in the case of the thin films containing a high amount of TaO_x_N_y_ (0.02, 0.04, 0.08, 0.20, 0.22 Pa partial pressure).

The spectra of the films produced with partial pressures in the range of 0.20–0.24 Pa are transparent to visible light and show characteristic interference fringes in the visible domain. One of the films containing higher amounts of metallic Ta (produced with partial pressure = 0.04 Pa) presents the highest absorption maximum among all the films, as was also found in previous research [40]. 

The UV-Vis spectra were also used to find the type of energy transition and the optical band gap of the Ta species, using the Tauc plots, considering that the optical absorption coefficient *α* depends on the photon energy (*hν*) according to Equation (2):

(2) $$\ln\left(\alpha h\upsilon \right)=ln\alpha_{0}+n\times \ln\left(h\upsilon -E_{g}\right)$$

where *α_0_* is the band tailing parameter and *n* is the power factor of the transition mode (for direct allowed transitions, *n* = 0.5, for the forbidden transitions *n* = 1.5, while for indirect allowed transitions, *n* = 2). To determine the type of transitions for each type of film, ln(*αhν*) was plotted as a function of *n* · ln(*hν* − *E_g_*), giving (for a certain region of the spectrum) a straight line with the slope equal to the power factor *n* [41]. By plotting (*αhν*)^1/2^, or (αhν)^2^, as a function of the photon energy, for indirect or direct transitions, respectively, the resulting dependency will have a linear part, whose intersection with the *hν* axis gives the values for the optical band gap energy, *E_g_* (eV) [42]. The valence band and the conduction band edge potentials (*E_VB_*, respectively *E_CB_*) have been calculated with Equation (3) and (4):

(3) $$E_{VB}=X-4.5+0.5\cdot E_{g}$$

(4) $$E_{CB}=X-4.5-0.5\cdot E_{g}$$

where *X* is the absolute electronegativity of the semiconductor species, determined according to Renuka et al. [43] as 5.47 for TaN and 5.98 for TaO_x_N_y_. The results are depicted in Table 2.

It can be seen from Table 2 that the *Eg* values of the thin films range from 1.43 to 2.67 eV. The reported values for *Eg* of tantalum oxynitrides typically range from 1.9 to 2.5 eV [44,45], while tantalum nitrides (TaN_x_) present optical band gaps in the 1.88–2.90 eV range. The *Eg* values for tantalum nitrides (when co-present with TaO_x_N_y_) tend to be slightly lower than in the case of TaO_x_N_y_ [46,47].

In the samples obtained at 0.13 and 0.17 Pa, where both the nitride and the oxynitride species are present in a significant amount (Table 2), two band gaps are observed, with the lower value being ascribed to TaN and the higher one being owed to TaO_x_N_y_. In the samples obtained with a 0.02, 0.04, 0.08, 0.20, 0.22, and 0.24 Pa partial pressure, where the oxynitride content is prevalent, only one Eg value has been registered. Nearly all the transitions are of an indirect type (n ≈ 2), while for the sample obtained at 0.24 Pa, a direct type of transition has been registered. The decreasing of the Eg values for these last three samples, as the partial pressure increases (and the O:N ratio, in the films composition, increases), could be due to the presence of lattice defects promoted by the higher oxygen content, as determined in other research [48].

For the chemical systems that present only one value of *E_g_* (other extrapolations of linear domains to the photon energy axis are forbidden, i.e., n ≈ 1.5), this value could be ascribed to tantalum oxynitride. It may not be ruled out that the nitride/oxynitride phases may form inter-associations (i.e., they may possibly form solid solutions in certain regions of the sample surface, with localized areas of a high density of stacking defects, possibly induced by Ta_2_O_5_) [48].

As localized states extended in the bandgap could often be found in disordered and amorphous materials, the Urbach energy (*E_U_*), i.e., the width of the tail of localized states in the bandgap, has been determined for each film type, through linearly extrapolating the ln(α) dependence on photon energies to low hν values [49]. The results depicted in Table 2 show an increase in the EU values with the increase in reactive gas flow, respectively TaO_x_N_y_ content, which could lead to an increase in the disordered atoms and in the defects in the structural bonding [50,51]. The lack of a significant rise in the reflectance values of the films above their Eg values may also show a high concentration of defect states in these materials [52].

### 3.2. Transmission Electron Microscopy (TEM)

**Figure 8:**
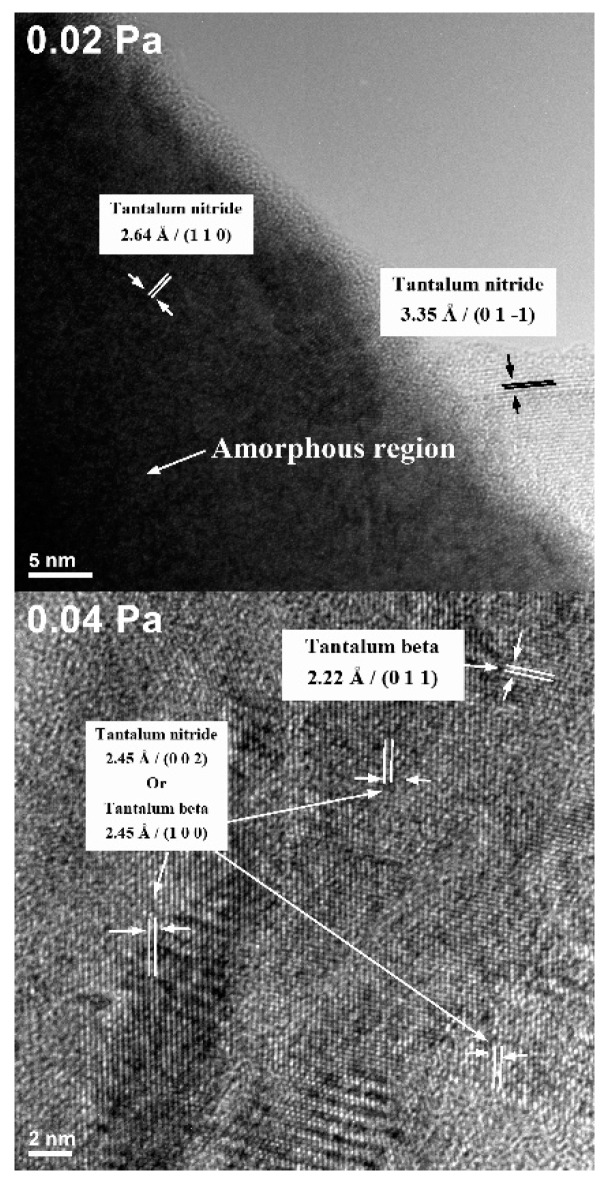
HRTEM images of the samples produced with *P*(N_2_ + O_2_) = 0.02 Pa and *P*(N_2_ + O_2_) = 0.04 Pa.

The HRTEM micrographs for the two representative samples which showed a good photodegradation ability (the ones deposited with 0.02 and 0.04 Pa reactive gas mixture partial pressure) are illustrated in Figure 8. These samples show the presence of both metallic Ta (β − Ta) and nitride phases. In the sample obtained at 0.02 Pa, both crystalline and amorphous regions could be seen. 

The average spacing between the crystallographic planes is 2.64 to 3.55 Å in the sample produced with *P*(N_2_ + O_2_) = 0.02 Pa, consistent with the TaN (110) and (01-1) crystallographic planes spacing. In the sample obtained at *P*(N_2_ + O_2_) = 0.04 Pa, several in-plane randomly-oriented domains could be observed, with possible alternation of crystalline and amorphous phases. In the case of the *P*(N_2_ + O_2_) = 0.04 Pa sample, the amorphous phase is significantly less present, and some grains correspond to β-Ta ((011) and (100) with 2.22 Å and 2.45 Å interplanar distance). The remaining samples, obtained with higher partial pressure values, are amorphous, as demonstrated by X-Ray Diffraction in our previous work [24].

### 3.3. Surface Morphology (AFM)

The surface morphology has a pronounced effect on the photocatalytic activity. For high photocatalytic activity, the thin film should have a high effective surface area, and thus an increased surface roughness. This increases the number of active sites and the number of defects. An increased surface roughness can be obtained by choosing a substrate with a rough surface, but can also be promoted by generating loosely packed small grains. Quantitative measurements of roughness and surface area, obtained using AFM, suggest that the deposition parameters do not significantly influence the surface roughness of the tantalum oxynitride thin films presented in this paper. 

**Figure 9:**
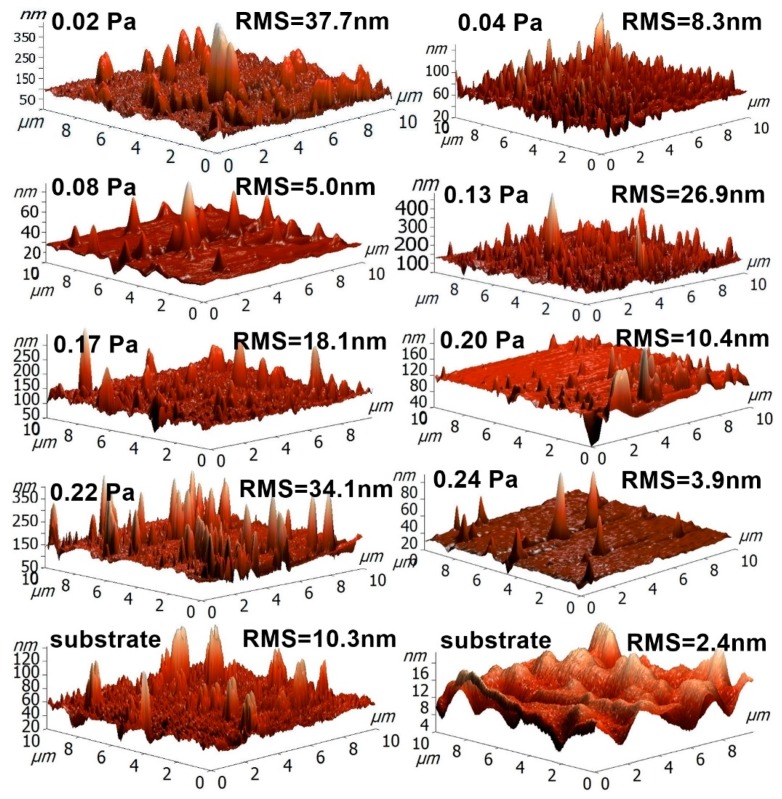
3D AFM images of TaO_x_N_y_ thin films deposited on glass substrates, with their respective RMS values.

Figure 9 presents the RMS roughness values for some of the as-deposited samples and uncoated substrates. The samples were analyzed on 10 × 10 μm^2^ areas. From the RMS roughness values, and also considering the surface features inherited from the substrate, one can conclude that the deposition process variable parameter (the reactive gas partial pressure) does not have a significant influence on the surface roughness, and, furthermore, that the preparation stage of the substrate could be used, at least in this particular case, to increase the effective surface area of the tantalum oxynitride films, due to the fact that the films follow the irregularities of the substrate surface. However, all the RMS roughness values can be relatively low, which translates into a relatively small effective surface area of the material. Higher surface roughness values would allow for a better adsorption of dye molecules on the surface of the films and subsequent a faster degradation of the molecules. Thus, the surface roughness would be one of the first parameters to be improved in order to obtain a higher photocatalytic activity for the tantalum oxynitride films deposited using the conditions presented herein.

### 3.4. Photodegradation Efficiency

**Figure 10:**
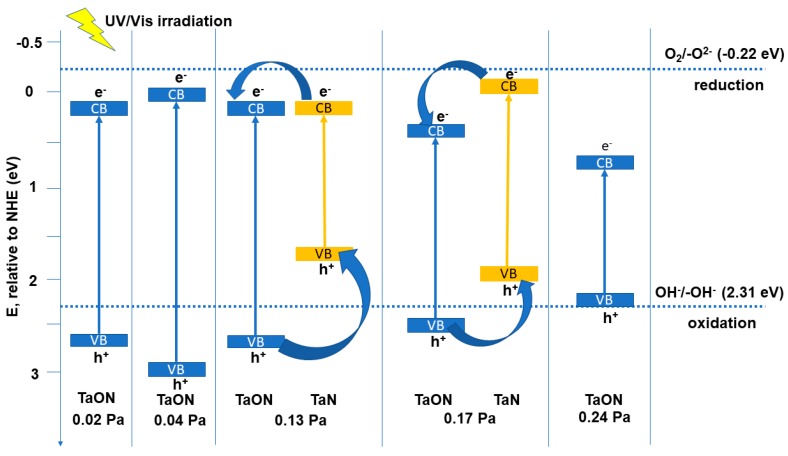
Band edge positions for the thin films.

All the films could act as visible light harvesters (due to their low *Eg* values), but, in their as-obtained form, the alignment of their conduction and valence bands is less favorable for the degradation of organic compounds (i.e., the conduction bands lie below the oxygen reduction potential at −0.22 eV, Figure 10), or due to fast recombination of the charge carriers [53,54]. 

The main photodegradation mechanism in this case would be the oxidation of the organic dyes with OH• radicals, generated from hydroxyl groups (-OH-) by photoneutralization with holes (h^+^) at the catalyst-solution interface, or through direct oxidation by a reaction with the photogenerated holes [55,56]:

(H_2_O ⇔ H^+^ + OH^−^) + h^+^ → H^+^ + OH• (free radicals photogeneration) (5)

Dye + OH• → R• + H_2_O (dye degradation) (6)

Dye + h^+^ → R^+^• → Degradation products (direct dye degradation) (7)

**Figure 11:**
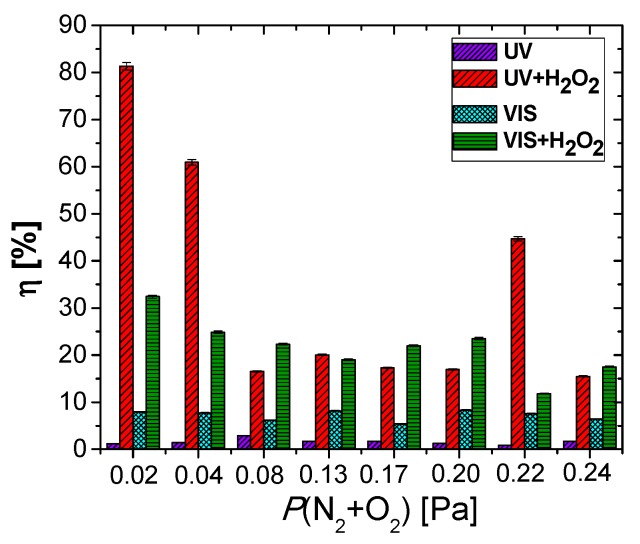
Variation of the photodegradation efficiency at equilibrium for the tantalum oxynitride thin films as a function of the partial pressure of the reactive gas.

**Figure 12:**
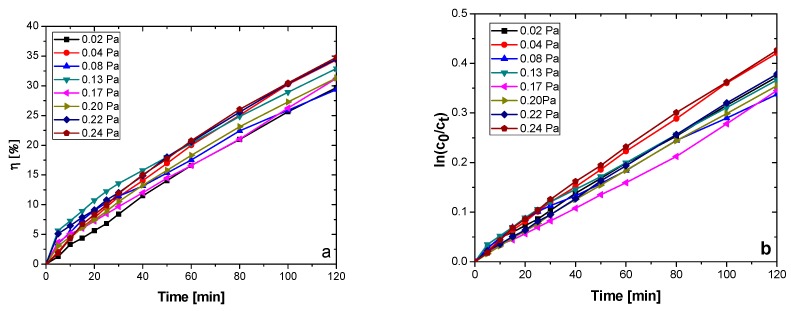
(**a**) Photodegradation efficiencies of the tantalum oxynitride samples; (**b**) graphical determination of the reaction rate of the photocatalytic degradation of MB.

Figure 11 exhibits the methylorange decolorization/degradation efficiency after 6 h of contact. The MO decolorization/degradation efficiency in the case of UV irradiation ranges from 1.16 to 1.74% (Figure 12), increasing with the amount of oxygen in the reactive gas flow, causing an increase in TaO_x_N_y_ content of the films (Figure 2). The use of this source of energy for photocatalysis in the case of the TaO_x_N_y_ films seems inefficient, due to the fast charge carriers’ recombination. It could be seen from Figure 11 that the films are able to use the visible light in a more efficient manner, a fact seen from the higher efficiencies (6.67 to 7.94%), per the optical band gaps of each system. The film obtained at 0.24 Pa has the lowest photodegradation efficiency, due to the unfavorable positioning of TaO_x_N_y_ valence and conduction band potentials. The highest VIS efficiency for MO degradation is reached for the samples obtained at 0.13 Pa and 0.20 Pa. In the case of the sample obtained with a 0.13 Pa partial pressure, a relatively high RMS roughness has been recorded compared to the remaining samples, as determined from the AFM measurements, which determines an increase in the specific surface area of this sample, when in contact with the methylorange aqueous solution. Additionally, a heterojunction between TaO_x_N_y_ and TaN could be seen (Figure 10), which may lead to a more efficient charge carriers separation. After hydrogen peroxide addition, the efficiency of the photodegradation process is increased, due to the decrease in the charge carriers recombination (H_2_O_2_ is an electron acceptor), as well as due to an increased production in hydroxyl radicals (H_2_O_2_ could be photoreduced by the electrons at the conduction band or it could photocleave by UV radiation) [57].

H_2_O_2_ + e^−^ → OH• + OH^−^ (8)

H_2_O_2 (h__ν__)_ → 2 OH• (9)

The addition of hydrogen peroxide to the aqueous MO solution has a significant influence on the photodegradation efficiency. Photodegradation efficiencies as high as ~82% after the UV irradiation, and close to 34% after VIS irradiation, can be observed in Figure 11. The highest performance in this case is exhibited by the lower partial pressure samples, namely the ones obtained with 0.02 and 0.04 Pa. For the 0.22 Pa partial pressure sample, the high photodegradation rate could be attributed to the slight increase in surface roughness (thus a bigger effective surface area), when compared, for example, with the samples obtained at the next lower and next higher partial pressures: *P*(N_2_ + O_2_) = 0.20 Pa − RMS = 10.4 nm, *P*(N_2_ + O_2_) = 0.22 Pa − RMS = 34.1 nm, *P*(N_2_ + O_2_) = 0.24 Pa − RMS = 3.9 nm (Figure 9). The sample obtained with a 0.24 Pa partial pressure presents one of the lowest photocatalytic activities, despite its narrow band gap. Being a direct semiconductor, the charge carriers’ recombination rate is higher than in the case of the other indirect semiconducting samples [58]. As it can be seen in Figure 12a, the VIS light-mediated methylene blue photodegradation efficiencies of the films range from 29 to 34%, while for the reference, the photodegradation efficiency was negligible (1.34%—not pictured).

The dependence of the methylene blue concentration on irradiation time has been modelled against the pseudo-first order kinetic model (simplification of the Langmuir–Hinshelwood mechanism for heterogenous photocatalysis), shown by Equation (10) in linearized logarithmic form [59]:

(10) $$\ln\frac{c_{t}}{c_{0}}=k\cdot t$$

where *c*_0_ represents the initial concentration of methylene blue (0.0125 mM), *c_t_* represents the MB concentration at different photoirradiation periods “*t*”, and *k* (min^−1^) is the pseudo-first-order rate constant.

**Figure 13:**
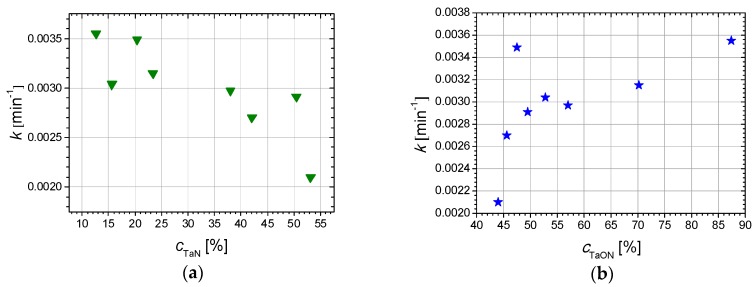
(**a**) Variation of the rate constant, *k*, of MB, with the concentration of TaN and (**b**) TaO_x_N_y_ phases in the films, detected by XPS.

It can be seen, from Table 3 and Figure 13, that the photodegradation rate depends on the TaN and TaO_x_N_y_ content and on the surface roughness of the films. The sample obtained at *P*(N_2_ + O_2_) = 0.24 Pa presents the highest photodegradation rate and highest MB photodegradation/discoloration efficiency from the analyzed sample set, correlated with the highest content in TaO_x_N_y_ (Figure 13). 

The samples obtained with *P*(N_2_ + O_2_) = 0.02 Pa and *P*(N_2_ + O_2_) = 0.04 Pa also exhibit high k values, due to the relatively high roughness, compared to the remaining samples (the former case) or high TaO_x_N_y_ content (the latter case). The samples with a higher content of Ta_2_O_5_ on the surface present the lowest photodegradation rates and lower photodegradation efficiencies, in accordance with other research [60]. 

Good wettability (decreased contact angle) is essential for a high photocatalysis reaction rate, ensuring a rapid equilibration of the contact angles at lower values, thus maximizing the contact surface between the photoactive surface and various diluted organic pollutant solutions.

**Figure 14:**
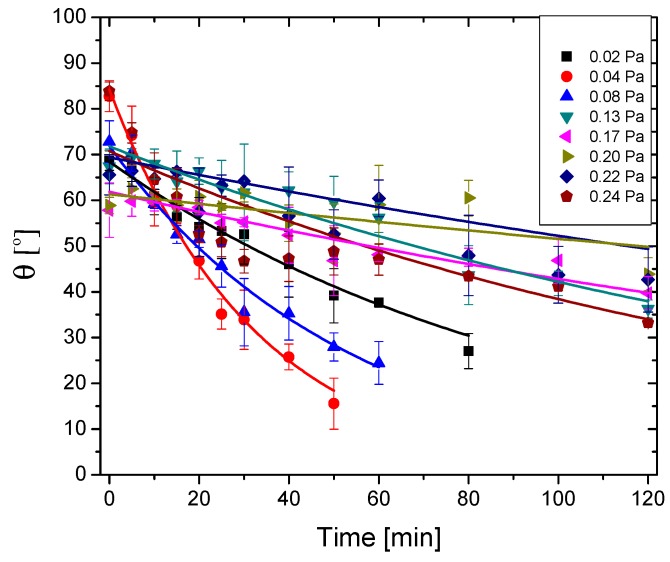
Contact angle kinetic of the films under UV irradiation.

As the RMS roughness of the films presents low values, the contact angles were not corrected for roughness. All the deposited films present a hydrophilic character, essential for photocatalysis in aqueous environments. The initial values of the contact angles (at the beginning of the wetting process) span between 61 and 86° (Figure 14), being significantly higher than in the case of the reference glass substrate (4.3°—not shown).

With the increase of the reactive gas flow, the films become less metallic and the contact angles tend to decrease, due to the formation of more hydrophilic oxygen-rich species on the surface of the samples (TaO_x_N_y_, Ta_2_O_5_). For all the films, the contact angles decrease considerably after UV irradiation (with 40–73%), possibly due to the formation of oxygen vacancies on the surface of the materials, which in turn can photocleave the adsorbed water molecules, leading to transient surface hydroxyl groups, of the Ta-OH type [61,62,63,64]. The contact angle kinetic was fitted against an exponential dependency of time, described by Equation (11):

(11) $$\theta =\theta_{0}\cdot e^{-k_{\theta }\cdot t}$$

where *θ*_0_ is the contact angle at the beginning of the wetting process (i.e., at *t* = 0) and *k_θ_* is the wetting rate of the surface (related to the drop spreading on the surface of the sample). The fitting parameters are expressed in Table 3. The highest wetting rates are recorded for the samples obtained with partial pressures of the reactive mixture of 0.02, 0.04, and 0.24 Pa, for which good photodegradation rates and efficiencies have been registered. This could imply a higher amount of hydroxyl groups formation on the surfaces of these samples, containing a higher amount of metallic Ta [52,65]. 

### 3.5. Antibacterial/Antibiofilm Capacity

**Figure 15:**
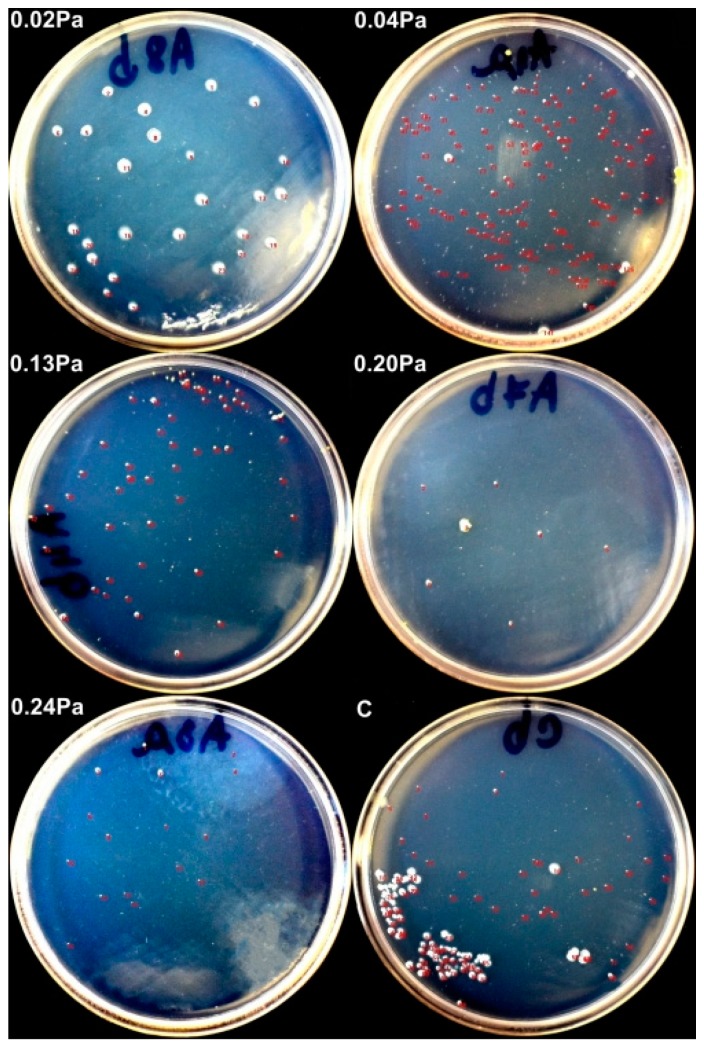
Bacterial colonies grown on agar plates.

Figure 15 exhibits results obtained on selected samples, concerning the antimicrobial/ antibiofilm capacity of the TaO_x_N_y_ coatings against *Salmonella*. During the incubation period of the samples covered with liquid media containing *Salmonella*, some of the grown bacterial cells have adhered to the surface of the samples. By rinsing the samples with PBS (Phosphate-buffered saline), the non-adherent bacteria have been removed. Following ultrasonication, the adherent bacteria has also been removed from the surface and immersed in PBS. 

**Figure 16:**
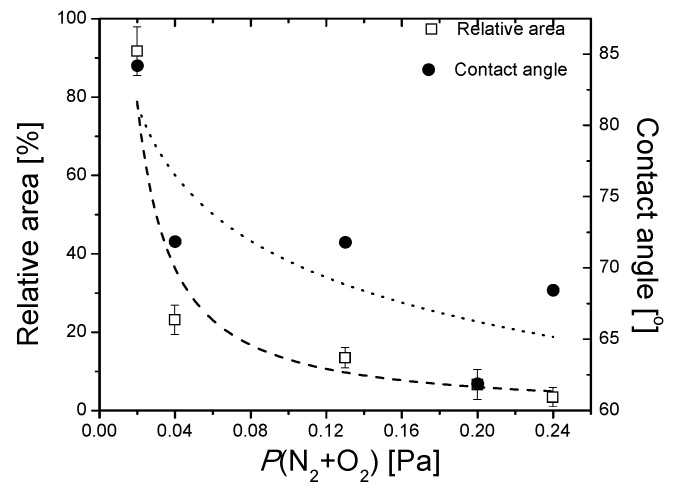
Relative surface area of the bacterial colonies and contact angle, as a function of the partial pressure.

After inoculation and incubation of the solution containing adherent bacteria, it could be observed that some bacterial colonies grew on agar plates after inoculation. The result of interest was the relative surface area of the bacterial colonies, compared to the control sample (uncoated AISI 316L steel). Evans and Sheel have reported that thin film coatings broaden the stainless-steel application field, due to their potential of improving the self-cleaning property of the material. This effect takes place due to a reduction in bacterial adhesion and colonization, which are mainly affected by the surface roughness [66,67]. On the other hand, some studies mention that the antimicrobial/antibiofilm potential of thin films could be negatively influenced by the oxide component [68,69]. Contrary to this observation, a higher antimicrobial/antibiofilm activity of the coatings was exhibited by the samples deposited with reactive mixture higher partial pressure, as shown in Figure 16. It has been shown that the antimicrobial performance of tantalum oxide coatings is predominantly exhibited by the amorphous structure, instead of the crystalline alternative, which has also outperformed human skin fibroblast cellular biocompatibility [22].

Chang and his co-authors have argued that a certain method which can ensure controlling bacterial adhesion on the coatings, because of the multitude of factors that influence the process, starting from surface properties to bacteria particularities, has not yet been developed [22]. If we consider the observation that tantalum does not have an intrinsic antibacterial activity, as demonstrated elsewhere [20], the results obtained on the sample deposited with the lowest partial pressure should be as expected. The potential causes for this behavior are the high RMS roughness (RMS = 37.7 nm), as well as the high metallic Ta content. 

Contrary to the expectations, the dependence between the relative surface area of the bacterial colonies and the contact angle is, in this case, inversely proportional. It was demonstrated that superhydrophobic surfaces (i.e., with higher contact angles) are much more suited to be used as antibacterial surfaces, due to their anti-adhesive capacity [70]. Anti-adhesive surfaces should reduce the adhesion force between bacteria and a solid surface to enable the easy removal of bacteria [71]. When discussing the surface roughness, higher values for Ra or RMS roughness do not necessarily mean better adhesion of the bacterial colonies, hence a poorer antibacterial capacity. Cells are easily removed from lower roughness surfaces, but they may be retained within features approximating in size to that of the cells. In larger features, the cells may again be relatively easily removed. Hence, the distance between surface features plays an important role. Micron-sized features may favor bacterial adhesion, whereas nano-sized features may create difficult surface conditions for attachment of the bacterial cells [72]. 

These observations are implying that there are more factors governing the antibacterial/antibiofilm activity. Even if the mechanism related to the antibacterial capacity is not entirely understood, tantalum-based (oxides) thin films have been tested, and they have antimicrobial potential against several microorganisms, such as *Staphylococcus aureus*, *Staphylococcus epidermidis*, *Actinobacillus actinomycetemcomitans*, and *Streptococcus mutans* [19,22]. The research in this area is necessary due to the reported high potential of tantalum to stimulate osseointegration. Moreover, if trabecular structures coated with Ta-based materials would be used, their geometry would make the adhesion of microorganisms to the surface difficult [20].

## 4. Conclusions

A set of tantalum oxynitride thin films were produced by DC reactive magnetron sputtering. Several configurations have been obtained by modifying the partial pressure of the reactive gases mixture (15% O_2_ + 85% N_2_) between 0.02 Pa and 0.24 Pa, while keeping the other deposition parameters identical (GND substrate, 100 °C substrate temperature, 1A target current, 1h deposition time, etc.).

X-ray Photoelectron Spectroscopy investigations highlighted tantalum film formation and surface chemical changes during reactive gas partial pressure exposure. The assessed tantalum chemical species provide information on the chemical reactions occurring at different deposition gas pressures. A dramatic decrease of metallic Ta was noticed with the increase of the reactive gas mixture partial pressure. Furthermore, a decreasing trend of nitride contribution accompanied by an increase of oxynitride feature was accomplished by increasing the deposition ambient partial pressure. The enhanced oxynitride content was associated with the favorable antibacterial properties of Ta films related to Salmonella. 

Transmission electron microscopy results from selected samples reveal both crystalline and amorphous regions, mainly composed of TaN and β-Ta. 

AFM measurements showed relatively low RMS roughness values, regardless of the deposition parameters, for the as-deposited samples. Photocatalytic experiments show promising results after dye photodegradation under several conditions: ultraviolet irradiation, with and without H_2_O_2_ added to the methyl orange solution; visible irradiation, with and without H_2_O_2_ added to the methyl orange solution. The photodegradation performance could be improved by increasing the sample roughness, either by changing the deposition parameters or using a high roughness substrate.

The antibacterial/antibiofilm capacity of the coatings was assessed against Salmonella. The result of interest was the relative surface area of the bacterial colonies, after inoculation, compared to the bare substrate. The coatings deposited with a higher reactive gas mixture partial pressure exhibit a significantly better antibiofilm capacity.

## Figures and Tables

**Table 1:** Deposition parameters for the tantalum oxynitride films.

Sample	Reactive Gas Flow (sccm)	Partial Pressure N_2_ + O_2_ (Pa)	Sputtering Pressure (Pa)
B2	2.5	0.02	0.41
B3	5	0.04	0.43
B4	10	0.08	0.47
B5	15	0.13	0.50
B6	20	0.17	0.54
B7	22.5	0.20	0.57
B8	25	0.22	0.59
B9	30	0.24	0.64

**Table 2:** Parameters associated with the Tauc plots. The column titled *E_U_* refers to Urbach energy.

Sample	*n*	*R* ^2^	*E_g_* (eV)		*E_U_* (meV)	Edge Potentials	
						*E_VB_* (eV)	*E_CB_* (eV)
0.02 Pa	1.863	0.996	2.265	TaO_x_N_y_	147.43	2.61	0.35
0.04 Pa	1.947	0.993	2.665	TaO_x_N_y_	175.67	2.81	0.15
0.08 Pa	1.817	0.964	2.508	TaO_x_N_y_	259.47	2.73	0.22
0.13 Pa	1.913	0.979	2.411	TaO_x_N_y_	182.19	2.68	0.27
	1.921	0.962	1.429	TaN		1.69	0.25
0.17 Pa	1.828	0.983	1.979	TaO_x_N_y_	331.61	2.46	0.49
	1.835	0.967	1.864	TaN		1.90	-0.03
0.20 Pa	2.008	0.997	1.956	TaO_x_N_y_	485.96	2.46	0.51
0.22 Pa	1.996	0.984	1.951	TaO_x_N_y_	484.16	2.45	0.50
0.24 Pa	0.501	0.970	1.436	TaO_x_N_y_	499.97	2.19	0.76

**Table 3:** MB photodegradation and contact angle kinetic parameters.

	MB Photodegradation Kinetic		Contact Angle Kinetic		
Sample	k (min^−1^)	R^2^	θ_0_ (°)	k_θ_ (min^−1^)	R^2^
0.02 Pa	0.00304	0.998	84.18	0.0303	0.982
0.04 Pa	0.00349	0.998	71.84	0.0185	0.977
0.08 Pa	0.00270	0.991	70.77	0.0062	0.942
0.13 Pa	0.00210	0.993	71.79	0.0054	0.972
0.17 Pa	0.00291	0.998	67.52	0.0030	0.960
0.20 Pa	0.00297	0.999	61.86	0.0032	0.879
0.22 Pa	0.00315	0.999	61.33	0.0017	0.865
0.24 Pa	0.00355	0.996	68.43	0.0101	0.990
